# From Mental Health to Mental Wealth in Athletes: Looking Back and Moving Forward

**DOI:** 10.3389/fpsyg.2016.00935

**Published:** 2016-06-21

**Authors:** Mark Uphill, Dan Sly, Jon Swain

**Affiliations:** Canterbury Christ Church UniversityCanterbury, UK

**Keywords:** flourishing, mental health, athlete, assets, mental illnesses

## Abstract

Considerations of athletes’ mental health are typically framed in the language of mental illness (Hughes and Leavey, 2012), a situation that contributes to stigmatization, denial, and the prevention of effective care. In this article, we provide a critical, narrative review of the extant literature on athlete mental health. Specifically, we begin by providing a brief synopsis of the extant literature on athletes’ mental health, illustrating both what we know about (i) the prevalence of mental health issues in sport and (ii) variables contributing to help-seeking behaviors in athletes. Against, this backdrop, we outline Keyes’ (2002) two-continuum model of mental health as a theoretical framework that has considerable promise in understanding, talking-about, and intervening to enhance, athletes’ mental health. This model posits two related, but distinct dimensions: one continuum indicates the presence or absence of mental health, the other the presence or absence of mental illness. From this perspective, a number of possibilities emerge. For instance, athletes could simultaneously have both positive mental health and experience of mental illness. Alternatively, athletes could be free from mental illness, but in Keyes’ terms be “languishing” (i.e., experiencing low levels of mental health). Implications for interventions based on the two-continuum model are discussed, particularly drawing on assets-based approaches to enhance flourishing (Theokas et al., 2005). We conclude the review by considering limitations in our understanding of how to promote flourishing and suggest avenues for further research.

## Introduction

Whether it is through media portrayals (Oldroyd, 2010), athlete autobiographies (Beard, 2012), mental health campaigns (Performance Matters), education (Thompson and Sherman, 2007), or academic research (Rao and Hong, 2016; Wolanin et al., 2016), there is an increased visibility of the mental health challenges experienced by athletes generally and elite sportspersons specifically (see Swann et al., 2015 for issues associated with the definition of “elite”). We use the term “athletes” in this paper to embrace the full spectrum of competitive sportspersons, and are more specific in our terminology where this is necessitated.

Against the increased prominence of the mental health challenges associated with elite sport in particular (Gulliver et al., 2015), is much unchartered territory in which (i) conceptions of mental health are widely contested (Rogers and Pilgrim, 2005), (ii) interventions to promote mental health should have a solid evidence-base (Jané-Llopis and Anderson, 2005), and (iii) there should be a better match between the priorities perceived by stakeholders (e.g., athletes, coaches, and policy makers) and those of the research community (Jané-Llopis and Anderson, 2005).

Just as there are different maps of the physical landscape, so the perspectives that are held regarding mental health can vary. Importantly, the way in which we conceive of mental health is not value-free (Cromby et al., 2013) – rather our conceptions are important practically, empirically, and politically. In this paper, we draw upon the two-continuum model of mental health (Keyes, 2002, 2005, 2007) as a framework that arguably has promise in understanding, talking about, and intervening to enhance, athletes’ mental health. In particular, we outline a growing body of empirical support for the model, and discuss the implications of understanding athletes’ mental health from this perspective.

We begin, however, by providing a summary of the extant literature on athletes’ mental health. Drawing upon descriptions of caricatures (Sparkes, 1992; Uphill and Dray, 2009), our intention is to highlight the key features of this literature, yet concomitantly obscure some of the subtleties and nuances that exist. The intention is not to cartoon the research or ideas of others, rather we use these central features to contextualize and strengthen our selection of this model.

## A Caricature of the Literature

Recent decades have witnessed a growth in research examining the spectrum of athletes’ mental health from mental illness (Gulliver et al., 2015), through to well-being (Lundqvist and Sandin, 2014). Nevertheless, this literature is characterized by differing ideas of what mental health “looks like” (cf. Rowley et al., 1995; Schwenk, 2000). Notwithstanding the challenges of arriving at a consensual definition, there are two themes associated with athletes’ mental health that are receiving research attention: (i) the prevalence of mental illness in elite athletes and (ii) barriers and facilitators of help-seeking among athletes more broadly.

With regard to the former, a cross-sectional survey of Australian elite athletes Gulliver et al. (2015), found that approximately 1 in 2 athletes (46%) were experiencing symptoms of at least one of the ‘mental health problems’ assessed, and the prevalence of problems was similar to that of community and athlete populations (e.g., Schaal et al., 2011). Although this study makes a valuable contribution to the literature, it is nonetheless symptomatic of the challenges confronting this endeavor. First, conclusions about the prevalence of mental ill-health are in part, shaped by the definitions and measures that are used. To illustrate as a point of contrast, Sundgot-Borgen and Torstveit (2004) suggested that the prevalence of eating disorders among Norwegian athletes, particularly for those athletes competing in sports that emphasize a lean body mass, was higher than in the general population.

Second, how mental health is assessed by researchers and practitioners may have different emphases. Research practices typically measure mental illness by virtue of a clinical or categorical score on a questionnaire or clinical interview (e.g., Gulliver et al., 2015), whereas in the UK at least, the practice-landscape draws upon constructions of mental health via a process of formulation (Kinderman and Tai, 2009) to navigate a terrain that draws upon clients’ experiences (Cromby et al., 2013) in promoting mental health.

With regards to help-seeking among athletes, although evidence suggests that elite athletes may be similar to the “general population” in terms of the prevalence of mental health difficulties, this is tempered with the recognition that stigma may be higher among athletes compared to non-athlete peers (Kaier et al., 2015). Stigma, coupled with a culture that emphasizes toughness and the minimisation of perceived weakness (Reardon and Factor, 2010) may contribute, in part, to under-recognition of mental illness in the athletic population.

Encouraging appropriate help-seeking by athletes then, is an important preventive and treatment strategy, yet athletes often do not seek professional help (Gulliver et al., 2012). A potential barrier to help-seeking among elite athletes is the [perceived] risk of admitting psychological difficulties that may result in exclusion from the team, being unable to compete, loss of livelihood, and athletic identity (Linder et al., 1989; Gulliver et al., 2012).

Acculturation of athletes may also influence help-seeking. For instance, Tan et al. (2014) suggest that the pursuit of thinness may be viewed positively within a sporting culture due to the advantages it holds to athletic success. The manner in which symptoms are labeled either by practitioners or athletes themselves may influence the degree of help-seeking. For example, athletes who may otherwise be described as experiencing depression could be labeled as ‘burnt-out’ due to similar presentations (Reardon and Factor, 2010). Similarly, athletes may have difficulty in distinguishing the fatigue and tiredness associated with training from depression (Schwenk et al., 2007). Additional barriers towards help-seeking include a lack of mental health literacy, and negative experiences of previous help-seeking (Gulliver et al., 2012). In contrast, facilitators of help-seeking include education and self-awareness, social support, and encouragement, positive relationships with practitioners and their integration into sporting life (Gulliver et al., 2012).

Third, we argue that there are comparatively few studies that have been directed towards the enhancement of athletes’ mental health. Some have attempted to reduce the stigmatization and likelihood of referral among athletes (e.g., Van Raalte et al., 2015), yet the evidence-base underpinning the promotion of mental health among athletes is poor.

### Summary

The caricature depicted above, is illustrative of a landscape for which much of the map remains to be drawn. In addition, the cartographer appears not to have a compass to help navigate the terrain. In short, there is little consensus about how best to conceptualize and assess athletes’ mental health, with little systematic progress about how best to promote athletes’ mental health. In the section below, Keyes’ (2002) two continuum model of mental health is proffered as a framework that may facilitate map-making (or what Forscher, 1963 described as edifice building) in the area of athletes’ mental health.

## Keyes’ (2002) Two Continuum Model

According to The World Health Organization [WHO] (2005, p. 2) mental health is not merely the absence of disease, it is “a state of well-being in which the individual realizes his or her own abilities, can cope with the normal stresses of life, can work productively and fruitfully, and is able to make a contribution to his or her community”. This understanding of mental health as more than the absence of mental illness is embraced within Keyes’ (2002) model in which mental health is characterized as a complete state. Specifically, Keyes developed the concept of a two continuum model; rather than see mental health and mental illness as residing at two ends of a single continuum, Keyes suggested that mental illness and mental health, are two distinct but related dimensions existing on two separate continua (see also Tudor, 1996). The first continuum relates to the absence and presence of mental illness, whilst the second pertains to the absence or presence of mental health. From this perspective, a number of possibilities emerge. For instance, athletes could simultaneously have both positive mental health and experience mental illness. Alternatively, athletes could be free from mental illness, but in Keyes’ terms be “languishing” (i.e., experiencing low levels of mental health).

There is a growing body of research supporting elements of this model (see Provencher and Keyes, 2011; Keyes, 2014 for reviews). Although space precludes a thorough consideration of this literature, a number of observations are pertinent. First, using latent measures of mental illness and mental health a two factor, rather than a single factor model, supported Keyes’ proposition that mental illness and mental health reside on two separate (albeit correlated) continua (Keyes’, 2002, 2005). In this research, major depressive order, panic, generalized anxiety disorder and alcohol dependence were diagnosed using the DSM– III-R (American Psychiatric Association, 1987). Using a similar approach, Keyes’ (2002) diagnosed mental health as a constellation of symptoms whereby, (i) flourishing was described as having high levels on at least one measure of hedonic well-being, and high levels on at least six measures of positive functioning, (ii) individuals who reported low levels of at least one measures of hedonic well-being and low levels in six measures of positive functioning were described as languishing, and (iii) moderately mentally healthy individuals did not fit the criteria described in (i) or (ii).

Secondly, research suggests less than perfect mental health is associated with increased impairment and disability (Keyes, 2002, 2005). Third, mental illness, when combined with languishing (i.e., low levels of mental health) is associated with greater impairment than when it occurs alongside moderate mental health or flourishing (Keyes and Michalec, 2010). Finally, Provencher and Keyes (2011) have conducted a systematic review of mental health literature, concluding that the adoption of a complete state model, facilitates restoration and optimisation following periods of ill health.

In short, there is accumulating evidence to suggest that the absence of mental health does not imply the presence of mental illness, and the presence of mental illness, does not imply the absence of mental health (see also Greenspoon and Saklofske, 2001; Suldo and Shaffer, 2008). Implications arising from this data suggest that interventions may be directed not only towards ameliorating mental illness, but actively promoting mental health. Indeed, and given the association between the two continua, the promotion of mental health is thought to reduce the propensity for developing mental illness. It is also evident that the two-continuum model is beginning to inform policy and practice (e.g., Friedli and Parsonage, 2009). In particular, strategies that are specifically directed towards promoting mental health (rather than reducing the incidence of mental illness), have adopted an assets-based approach to intervention (e.g., Foot, 2012).

A [mental] health asset, can be defined as,

“any factor (or resource), which enhances the ability of individuals, groups, communities, populations, social systems and/or institutions to maintain and sustain health and well-being and to help to reduce health inequities. These assets can operate at the level of the individual, group, community, and/or population as protective (or promoting) factors to buffer against life’s stresses” (Morgan and Ziglio, 2007, p. 18).

According to Friedli and Parsonage (2009) asset-based practice aim to (i) strengthen and promote variables that support good health and wellbeing, (ii) protect against poor health, and (iii) foster communities and networks that sustain health. Broadly speaking, assets-based approaches typically involve re-framing current thinking towards assets-based ideas, mapping of assets, understanding how assets can be connected and used, and a co-production of outcomes by professionals and individuals, such as athletes (Friedli and Parsonage, 2009). As Foot (2012) reviews, there is a growing body of empirical evidence supporting assets-based approaches to health promotion broadly, and mental health specifically. Despite much conceptual overlap between assets-based, and strengths-based approaches, the former is arguably broader in its focus (embracing both individual and community strengths), whereas the latter has tended to focus more on individual-level strengths and interventions (Boiler et al., 2013). To date, an assets-based approach has not been extended to the mental health of athletes.

Notwithstanding these encouraging developments, the two-continuum model advocated by Keyes (see Keyes, 2012, 2014 for reviews) is characterized by some limitations. In the following section we address those limitations and consider the implications of Keyes’ model for the promotion of mental health in athletes

## Implications and Limitations of Keyes’ Model for Promotion of Mental Health in Athletes

### Conceptualizing Athletes’ Mental Health

Thus far, we have deliberately sidestepped defining athletes’ mental health. Yet we would be remiss if we were not to consider the definition of mental health as it relates to Keyes’ model, and the implications for athletes. Considering Keyes’ model, it is apparent that the language of mental illness is stigmatizing for athletes, and that the diagnosis of mental illnesses have been seriously challenged (Rogers and Pilgrim, 2005). Moreover, just as there are challenges associated with diagnosing mental illness, so too are there challenges with the assessment of mental health (Friedli, 2009). Thus, the traditional language associated with Keyes’ model represents one limitation to it’s’ applicability to athletes.

Perhaps the real value of the Keyes’ model then is not so much in the labels attached to the two-continua, but rather the perspective that it affords (Walker, 2006). Others have replaced mental illness or psychopathology with distress (Cromby et al., 2013), and for us, adopting a discourse that recognizes that athletes’ experiences that may fall along a continuum from “hardly distressing” to “extremely distressing” may help alleviate some of the stigma that may be associated with talking about mental illness. Similarly, recognizing that athletes may experience low levels of mental well-being at one end of the continuum and mental *wealth*, or flourishing at the other end, seems to us indicative of a situation in which we *all* have mental health needs (Friedli, 2009) and is less alienating and threatening than the dominant discourse. Thus *mental health* provides a conceptual space that encapsulates the broad spectrum of both distressing and flourishing experiences, but recognizes that the strategies designed to ameliorate distressing symptoms may not necessarily be the same as those designed to enhance flourishing.

### Assessment of Mental Health

Enhancement of mental health is to some degree predicated on ideas about how we understand and assess mental health. In Keyes’ terms, where self-reports of ‘mental health’ are coupled with self-reports of ‘mental illness’ different groups have been proposed. While the terminology, thresholds and measures implemented to describe differing groups have varied (cf., Suldo and Shaffer, 2008; Eklund et al., 2011) recognizing both continua in how we come to understand and improve athletes’ mental health is arguably valuable. That is, “well-adjusted” (high mental health, low mental illness), “symptomatic but content” (high mental health, high mental illness) “ambivalent” (low mental health, low mental illness) or “vulnerable” (low mental health, high mental illness), individuals may benefit from different interventions. From this vantage point, “traditionally” the needs of asymptomatic individuals who are in Keyes’ terms languishing may be overlooked (Eklund et al., 2011). Ambivalent individuals (i.e., who may be asymptomatic, yet experience low mental health) may be at risk for developing future mental illness (Keyes, 2012). A further limitation, not so much of the model, but of the measures used to examine its applicability across different contexts, is that appropriate instruments to facilitate such an assessment have yet to be developed in sport. A conceptualisation and assessment of mental health in athletes, grounded in Keyes’ two-continuum model, but drawing on *athletes’ experiences*, perhaps represents a pragmatic alliance between theory and practice and would be in accord with mental health users who have described themselves as *experts by experience* (Cromby et al., 2013).

### Interventions

Keyes’ (2012, 2014) model implies that the promotion of athletes’ mental health requires two complementary strategies: one directed toward the reduction and prevention of mental distress, the second towards the development and protection of flourishing. Assets-based approaches that draw on and develop individuals’ and organizations’ strengths may have some utility in the latter regard (Theokas et al., 2005). From an individual perspective when assets are assessed and developed, clients may be more likely to experience the intervention as empowering and motivating (Tedeschi and Kilmer, 2005). From an organizational perspective, focussing on organizational assets facilitated cultural change towards managing individuals’ mental health (Clossey et al., 2011). One important debate regarding mental health promotion concerns the balance between interventions that may strengthen the resilience of individuals, and those that impact wider determinants of mental health (Friedli, 2009). Much remains to be learned about the effectiveness, efficacy, and cost effectiveness of different mental health promotion strategies among athletes.

## Conclusion

In outlining the two continuum model of mental health, we have provided a conceptual space that addresses the full spectrum of athletes’ mental health, and in turn can impact our understanding of the antecedents and consequences of mental health for athletes. Moreover, the limitations and implications of Keyes’ model identified provides an impetus for further research in this area.

The concept of “mental health” from a linguistic perspective is that of an abstraction defined by clusters of “symptoms” – when we speak as if someone *has* a diagnosis or *has* a “mental illness” we are [unwittingly perhaps] creating a reality (Walker, 2006). And while the mental illness and mental health approach to clustering symptoms used by Keyes represents one perspective, arguably this need not be the only one. And thus what we offer here, is a perspective – a perspective in which our own [“professionals”] place in influencing understanding of athletes’ mental health is apparent. According to Anderson (1997, p. 71),

“…the culturally designated professional voice, usually speaks and decides for marginal populations…whether therapy is indicated and, if so, which therapy and toward what purpose. Sometimes unwittingly, sometimes knowingly, therapists subjugate or sacrifice a client to the influences of this broader context…”.

In sum, we all have one condition, the *human condition* marked by occasional, fluctuating, sometimes chronic, struggles with our thoughts, feelings, impulses, and habits, coupled with occasional, sometimes long-term sense of purpose, meaningfulness and well-being. Words are tools, not truths, and Keyes’ “tool” (compass?) may help us navigate our understanding of, and intervening to enhance, athletes’ mental health.

## Author Contributions

This article is derived from DS’s doctoral studies for which MU and JS are co-supervisors. We jointly conceived of the article and its focus. MU and DS initially drafted an outline of the article, upon which JS provided feedback, and suggested some alterations in focus and content. MU and DS each amended sections of the manuscript, before MU edited a penultimate draft of the manuscript. DS and JS both read this manuscript and approve of its contents.

## Conflict of Interest Statement

The authors declare that the research was conducted in the absence of any commercial or financial relationships that could be construed as a potential conflict of interest.

## References

[B1] American Psychiatric Association (1987). *Diagnostic and Statistical Manual of Mental Disorders* 3rd Edn. Washington, DC: Author.

[B2] Anderson H. (1997). *Conversation, Language, and Possibilities: a Postmodern Approach to Therapy.* New York, NY: Basic Books.

[B3] Beard A. (2012). *In The Water They Can’t See You Cry: A Memoir.* London: Simon & Schuster.

[B4] Boiler L. Haverman M. Westerhof G. J. Riper H. Smit F. Bohlmeijer E. (2013). Positive psychology interventions: a meta-analysis of randomised controlled studies. *BMC Public Health* 13:119 10.1186/1471-2458-13-119 PMC3599475 23390882

[B5] Clossey L. Mehnert M. Silva S. (2011). Using appreciative inquiry to facilitate implementation of the recovery model in mental health agencies. *Health Soc. Work* 36 259–266. 10.1093/hsw/36.4.259 22308878

[B6] Cromby J. Harper D. Reavey P. (2013). *Psychology, Mental Health and Distress.* London: Palgrave MacMillan.

[B7] Eklund K. Dowdy E. Jones C. Furlong M. (2011). Applicability of the dual-factor model mental health for college students. *J. Coll. Stud. Psychother.* 25 79–92. 10.1080/87568225.2011.532677

[B8] Foot J. (2012). *What Makes us Healthy? The Asset Approach in Practice: evidence, Action, and Evaluation.* Available at : http://www.janefoot.co.uk/downloads/files/healthy%20FINAL%20FINAL.pdf [accessed December, 2015].

[B9] Forscher B. K. (1963). Chaos in the brickyard. *Science* 142 339 10.1126/science.142.3590.339 17799464

[B10] Friedli L. (2009). “Future directions in mental health promotion and public health,” in *The Art And Science Of Mental Health Nursing* eds Norman I. Ryrie I. (London: McGraw-Hill) 43–61.

[B11] Friedli L. Parsonage M. (2009). *Promoting Mental Health and Preventing Mental Illness: the Economic Case For Investment In Wales.* Available at: http://www.publicmentalhealth.org/Documents/749/Promoting%20Mental%20Health%20Report%20 [accessed December, 2015].

[B12] Greenspoon P. J. Saklofske D. H. (2001). Toward integration of subjective well-being and psychopathology. *Soc. Indic. Res.* 54 81–108. 10.1023/A:1007219227883

[B13] Gulliver A. Griffiths K. M. Christensen H. (2012). Barriers and facilitators to mental health help-seeking for young elite athletes: A qualitative study. *BMC Psychiatry* 12:157 10.1186/1471-244X-12-157 PMC3514142 23009161

[B14] Gulliver A. Griffiths K. M. Mackinnon A. Batterham P. J. Stanimirovic R. (2015). The mental health of Australian elite athletes. *J. Sci. Med. Sport* 18 255–261. 10.1016/j.jsams.2014.04.006 24882147

[B15] Hughes L. Leavey G. (2012). Setting the bar: athletes and vulnerability to mental illness. *Br. J. Psychiatry* 200 95–96. 10.1192/bjp.bp.111.095976 22297587

[B16] Jané-Llopis E. Anderson P. (2005). *Mental Health Promotion and Mental Disorder Prevention. A policy for Europe.* Nijmegen: Radboud University Nijmegen.

[B17] Kaier E. DeMarni Cromer L. Johnson M. D. Strunk K. Davis J. L. (2015). Perceptions of mental illness stigma: comparisons of athletes to non-athlete peers. *J. Coll. Stud. Dev.* 56 735–739. 10.1353/csd.2015.0079

[B18] Keyes C. (2007). Promoting and protecting mental health as flourishing: a complementary strategy for improving national mental health. *Am. Psychologist* 62 95–108. 10.1037/0003-066X.62.2.95 17324035

[B19] Keyes C. L. M. (2002). The mental health continuum: from languishing to flourishing in life. *J. Health Soc. Behav.* 43 207–222. 10.2307/3090197 12096700

[B20] Keyes C. L. M. (2005). Mental illness and/or mental health? Investigating axioms of the complete state model of health. *J. Consult. Clin. Psychol.* 73 539–548. 10.1037/0022-006X.73.3.539 15982151

[B21] Keyes C. L. M. (2012). “Toward a science of mental health,” in *The Oxford Handbook Of Positive Psychology* 2nd Edn eds Lopez S. J. Snyder C. R. (Oxford: Oxford University Press) 89–95.

[B22] Keyes C. L. M. (2014). “Mental health as a complete state: how the salutogenic perspective completes the picture,” in *Bridging Occupational, Organisational, And Public Health: A Transdisciplinary Approach* eds Baur G. F. Hämmig O. (London: Springer) 179–187.

[B23] Keyes C. L. M. Michalec B. (2010). “Viewing mental health from the complete state paradigm,” in *A Handbook for The Study Of Mental Health: Social Contexts, Theories, and System* 2nd Edn eds Sheid T. L. Brown T. (New York, NY: Cambridge University Press) 125–134.

[B24] Kinderman P. Tai S. (2009). *Psychological Health and Well-Being: A New Ethos for Mental Health. A Report of the Working Group on Psychological Health and Well-Being.* Leicester: British Psychological Society.

[B25] Linder D. E. Pillow D. R. Reno R. R. (1989). Shrinking jocks: derogation of athletes who consult a sport psychologist. *J. Sport Exerc. Psychol.* 11 270–280.

[B26] Lundqvist C. Sandin D. (2014). Well-being in elite sport: dimensions of hedonic and eudaimonic well-being among elite orienteers. *Sport Psychologist* 28 245–254. 10.1123/tsp.2013-0024

[B27] Morgan A. Ziglio E. (2007). Revitalising the evidence base for public health: an assets model. *Glob. Health Promot.* 14 17–22. 10.1177/10253823070140020701x 17685075

[B28] Oldroyd E. (2010). *The Pressure to Hide Depression.* Retrieved on 09/12/2015 from http://www.bbc.co.uk/programmes/b00vv0dx

[B29] Provencher H. L. Keyes C. L. M. (2011). Complete mental health recovery: bridging mental illness with positive mental health. *J. Public Ment. Health* 10 57–69. 10.1108/17465721111134556

[B30] Rao A. L. Hong E. S. (2016). In the mood for change: shifting the paradigm of mental health care in athletes – an AMSSM thematic issue. *Br. J. Sports Med.* 50 133–134. 10.1136/bjsports-2015-095924

[B31] Reardon C. L. Factor R. M. (2010). Sport psychiatry a systematic review of diagnosis and medical treatment of mental illness in athletes. *Sports Med.* 40 961–980. 10.2165/11536580-000000000-00000 20942511

[B32] Rogers A. Pilgrim D. (2005). *A Sociology of Mental Health and Illness.* Berkshire: Open University Press.

[B33] Rowley A. J. Landers D. M. Kyllo L. B. Etnier J. L. (1995). Does the iceberg profile discriminate between successful and less successful athletes – a meta-analysis. *J. Sport Exerc. Psychol.* 17 185–199.

[B34] Schaal K. Taﬄet M. Nassif H. Thibault V. Pichard C. Alcotte M. (2011). Psychological balance in high level athletes: gender-based differences and sport-specific patterns. *PLoS ONE* 6:e19007 10.1371/journal.pone.0019007 PMC3087722 21573222

[B35] Schwenk T. (2000). The stigmatisation and denial of mental illness in athletes. *Br. J. Sports Med.* 34 4–5. 10.1136/bjsm.34.1.4 10690441 PMC1724145

[B36] Schwenk T. L. Gorenflo D. W. Dopp R. R. Hipple E. (2007). Depression and pain in retired professional footballers. *Med. Sci. Sports Exerc.* 39 599–605. 10.1249/mss.0b013e31802fa679 17414796

[B37] Sparkes A. C. (1992) “The paradigms debate: an extended review and a celebration of difference,” in *Research In Physical Education and Sport: Exploring Alternative Visions* ed. Sparkes A. C (London: Falmer Press) 9–60.

[B38] Suldo S. M. Shaffer E. J. (2008). Looking beyond psychopathology: the dual-factor model of mental health in youth. *School Psych. Rev.* 37 52–68.

[B39] Sundgot-Borgen J. Torstveit M. K. (2004). Prevalence of eating disorders in elite athletes is higher than in the general population. *Cl. J. Sports Med.* 14 25–32. 10.1097/00042752-200401000-00005 14712163

[B40] Swann C. Moran A. Piggott D. (2015). Defining elite athletes: issues in the study of expert performance in sport psychology. *Psychol. Sport Exer.* 16 3–14. 10.1016/j.psychsport.2014.07.004

[B41] Tan J. Bloodworth A. McNamee M. Hewitt J. (2014). Investigating eating disorders in elite gymnasts: conceptual, ethical and methodological issues. *Eur. J. Sport Sci.* 14 60–68. 10.1080/17461391.2012.728632 24533496

[B42] Tedeschi R. G. Kilmer R. P. (2005). Assessing strengths, resilience, and growth to guide clinical interventions. *Prof. Psychol. Res. Pr.* 36 230–237. 10.1037/0735-7028.36.3.230

[B43] Theokas C. Almerigi J. B. Lerner R. M. Dowling E. M. Benson P. L. Scales P. C. (2005). Conceptualizing and modelling individual and ecological asset components of thriving in early adolescence. *J. Early Adolesc.* 25 113–143. 10.1177/0272431604272460

[B44] Thompson R. A. Sherman R. T. (2007). *Managing Student-Athletes Mental Health Issues. National Collegiate Athletic Association.* Available at: http://www.wellawaresp.org/pdf/112012/2007NCAA_managing_mental_health.pdf

[B45] Tudor K. (1996). *Mental Health Promotion: Paradigms and Practice.* London: Routledge.

[B46] Uphill M. A. Dray K. (2009). Giving yourself a good beating: appraisal, attribution, rumination and counterfactual thinking. *J. Sports Sci. Med.* 8 5–12. 24474879 PMC3879640

[B47] Van Raalte J. L. Cornelius A. E. Andrews S. Diehl N. S. Brewer B. W. (2015). Mental health referral for student-athletes: Web-based education and training. *J. Clin. Sport Psychol.* 9 197–212. 10.1123/jcsp.2015-0011

[B48] Walker M. T. (2006). The social construction of mental illness and its implications for the recovery model. *Int. J. Psychosoc. Rehabil.* 10 71–87.

[B49] Wolanin A. Hong E. Marks D. Panchoo K. Gross M. (2016). Prevalence of clinically elevated depressive symptoms in college athletes and differences by gender and sport. *Br. J. Sports Med.* 50 167–171. 10.1136/bjsports-2015-095756 26782764

[B50] World Health Organization [WHO] (2005). *Promoting Mental Health: Concepts, Emerging Evidence, Practice. WHO in Collaboration With the Victorian Health Promotion Foundation and University of Melbourne.* Geneva: WHO Press.

